# A review of mechanistic learning in mathematical oncology

**DOI:** 10.3389/fimmu.2024.1363144

**Published:** 2024-03-12

**Authors:** John Metzcar, Catherine R. Jutzeler, Paul Macklin, Alvaro Köhn-Luque, Sarah C. Brüningk

**Affiliations:** ^1^ Intelligent Systems Engineering, Luddy School of Informatics, Computing, and Engineering, Bloomington, IN, United States; ^2^ Informatics, Luddy School of Informatics, Computing, and Engineering, Bloomington, IN, United States; ^3^ Department of Health Sciences and Technology (D-HEST), Eidgenössische Technische Hochschule Zürich (ETH), Zürich, Switzerland; ^4^ Swiss Institute of Bioinformatics (SIB), Lausanne, Switzerland; ^5^ Oslo Centre for Biostatistics and Epidemiology, Faculty of Medicine, University of Oslo, Oslo, Norway; ^6^ Oslo Centre for Biostatistics and Epidemiology, Research Support Services, Oslo University Hospital, Oslo, Norway

**Keywords:** mathematical modeling, machine learning, deep learning, ODE (ordinary differential equation), mechanistic learning

## Abstract

Mechanistic learning refers to the synergistic combination of mechanistic mathematical modeling and data-driven machine or deep learning. This emerging field finds increasing applications in (mathematical) oncology. This review aims to capture the current state of the field and provides a perspective on how mechanistic learning may progress in the oncology domain. We highlight the synergistic potential of mechanistic learning and point out similarities and differences between purely data-driven and mechanistic approaches concerning model complexity, data requirements, outputs generated, and interpretability of the algorithms and their results. Four categories of mechanistic learning (sequential, parallel, extrinsic, intrinsic) of mechanistic learning are presented with specific examples. We discuss a range of techniques including physics-informed neural networks, surrogate model learning, and digital twins. Example applications address complex problems predominantly from the domain of oncology research such as longitudinal tumor response predictions or time-to-event modeling. As the field of mechanistic learning advances, we aim for this review and proposed categorization framework to foster additional collaboration between the data- and knowledge-driven modeling fields. Further collaboration will help address difficult issues in oncology such as limited data availability, requirements of model transparency, and complex input data which are embraced in a mechanistic learning framework

## Graphical Abstract

Data and knowledge both drive the progress of research and are the cornerstones of modeling. Depending on the emphasis, both data-driven (exemplified by machine and deep learning) and knowledge-driven (exemplified by mechanistic mathematical modeling) models generate novel results and insights. Mechanistic learning describes approaches that employ both data and knowledge in a complimentary and balanced way.

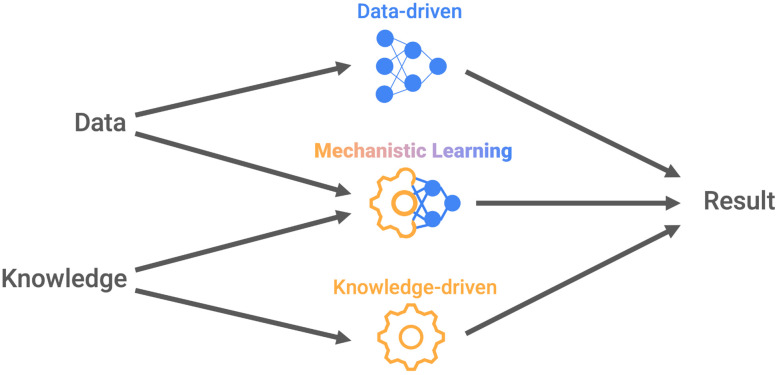

## 1 Introduction

An increasing understanding of cancer evolution and progression along with growing multi-scale biomedical datasets, ranging from molecular to population level, is driving the research field of mathematical oncology (1). Mathematical oncology aims to bridge the gaps between medicine, biology, mathematics, and computer science to advance cancer research and clinical care. Both data and understanding of cancer biology contribute to this aim. Furthermore, modeling in the context of clinical application poses a range of challenges that need to be met in order to ensure practical translation: data sparsity, heterogeneity, and source bias need to be accounted for, while the complexity of the model has to remain balanced regarding flexibility, interpretability, and explainability. Finally, one must consider the risk of model overfitting, together with robustness and generalization strength.

Data science may be defined as “*a set of fundamental principles that support and guide the principled extraction of information and knowledge from data*” *(* 2). Here, problem-solving is approached from the perspective of a learning process accomplished through observing diverse examples (3). Relationships between various types of input data (e.g., omics and imaging) and outcomes (e.g., overall survival) are abstracted where a mechanistic understanding of a relationship is missing or otherwise not accounted for. In this context, we refer to it as “data-driven” modeling. For oncology, data-driven approaches address a variety of applications to further scientific progress and task automation. Prime examples include predictions of drug response, tumor subtyping, and outcome as well as auto-segmentation of tumors on imaging.

An alternative is to formulate a specific guess on how relevant variables interact between input and output through the formulation of a mathematical model. Bender defines a mathematical model as an “*abstract, simplified, mathematical construct related to part of reality and created for a particular purpose*” *(* 4). Here the formulation of deliberate approximations of reality through equations or rules is key (5). In turn, the quality and limits of this approximation, which we refer to as “knowledge-driven” modeling, are validated with data. Independent of the use of a data science or a mathematical modeling formulation, “data” and “knowledge” are indispensable. The emphasis on data and knowledge may vary leading to the terminology of “data-driven” and “knowledge-driven” modeling (6). The fluid boundaries between these concepts motivate their combination.

The evolving field of mechanistic learning (7, 8) aims to describe synergistic combinations of classical mathematical modeling and data science (9, 10). In this review, we provide an overview of the key aspects of these approaches, explain possible ways of combining them, present a selection of examples, and discuss how mechanistic learning can thrive in mathematical oncology. In doing so, we aim to draw awareness to similarities and synergies between knowledge- and data-driven modeling, noting that this combination could help push mathematical oncology into the clinic as reliable, data-supported, and explainable models in the context of oncology (11).

## 2 Contrasting “knowledge-driven” and “data-driven” modeling”

As per definition, data- and knowledge-driven modelling are complementary perspectives for approaching research questions. Here, we address similarities and differences to understand synergies at the interface of these fluid concepts.

### 2.1 Knowledge-driven modeling approximates biomedical understanding

According to Rockne et al. (1), the goal of knowledge-driven modeling is to describe the behavior of complex systems based on an understanding of the underlying mechanisms rooted in fundamental principles of biology, chemistry, and physics. While the formulation of the “model”, i.e. the approximation of reality, is flexible, the overarching aim is to gain a deeper understanding of processes driving the system’s behavior often through simulation and analysis of unobserved scenarios. Here, mathematical formulas or systematic processes are purposefully crafted to reflect key aspects of reality with inevitable simplifying assumptions. For example, dimensionality is reduced, dynamic processes are approximated as time-invariant, or biological pathways are reduced to key components (12). Conceptualizing these assumptions requires a deep understanding of the biomedical processes and modeling goals. These demands are met through interdisciplinary collaboration and validation. In the absence of experimental data, it is still possible to analyze and simulate to expose dynamics emerging from model building blocks (13–15). These extrapolations beyond the range of validation data are rooted in the confidence in the quality of the approximation of the biomedical reality, i.e. the quality of the knowledge and its implementation.

It is tempting to suggest that knowledge-driven models are inherently interpretable. Yet, the implementation of chains of relationships can formulate complex inverse problems. Subsequently, *post hoc* processing through parameter identifiability and sensitivity analyses is key (16, 17). This can identify previously unknown interactions between system components to generate hypotheses for experimental and clinical validation.

Knowledge-driven modeling has successfully been applied to investigate different aspects of cancer including somatic cancer evolution and treatment. We refer the interested reader to recent review articles (18, 19) covering for instance different fractionation schemes for radiotherapy (20, 21), the onset and influence of treatment-induced tumor resistance (22), or cancer evolution (23). A popular application of knowledge-driven models is the simulation of *in silico* trials for hypothesis generation in simulated cohorts (24–26).

### 2.2 Data-driven models extract information from data

A common understanding of data-driven modeling (e.g. - machine learning, deep learning and classical statistics) is the creation of insight from empirical examples (27). A performance metric (28, 29) is optimized to uncover patterns and relationships between input data and output task. The validity of data-driven models should be studied carefully, in particular the dependency of the results on the chosen performance metric (29). It is also key to consider the optimization convergence. If this process fails, the model will be uninformative.

Purely data-driven models do not readily leverage the community’s understanding of the system under study but instead often employ highly parameterized models. The many degrees of freedom allow flexibility to approximate complex and mechanistically unknown relationships, e.g. deep neural networks act as “universal function approximators’’ (30). New information can be extracted from the data through this structuring but the extensive parameterization may obscure how the decision process is formed. *Post hoc* processing is required to uncover the nature of the approximated relationship through interpretability and explainability analysis (31). The models’ flexibility also makes them vulnerable to overfitting. Appropriately large amounts of training data and stringent data splits for fitting (training) and validation (32) are necessary to mitigate this risk. Data quantity and quality, i.e. its task specificity and ability to cover a variety of relevant scenarios, are equally important.

Generally, the application focus differs from that of knowledge-driven models. Generalization beyond the observed data space is often challenging (33). It is essential to rely on robust training regimes (34) and consider model limitations as performance is compromised in scenarios not (sufficiently) covered by data (33).

In summary, data-driven approaches are powerful tools for knowledge generation. In oncology, data-driven approaches have previously contributed substantially to scientific progress and process automation (35). To name just a few examples, (un-)supervised machine learning has greatly supported areas of drug response prediction (36, 37) and molecular tumor subtype identification (38, 39), whereas generative models and deep learning have revolutionized computer vision tasks such as volumetric tumor segmentation (40, 41), image-based outcome predictions (42, 43) and automated intervention planning.

### 2.3 Identifying similarities and boundaries between knowledge-driven and data-driven modeling

**Table 1** summarizes and contrasts key characteristics of the extremes of purely data- and knowledge-driven modeling, yet boundaries between these models remain fluid for many applications. The fundamental steps of data- and knowledge-driven modeling have parallels despite varying terminology: a subset of data is used to construct and calibrate the model, then further data is necessary for validation and refinement. In data-driven modeling, we first formulate the learning task (i.e. identifying features, labels, and loss function), and architecture selection. In knowledge-driven modeling, we start by deriving equations/mathematical rules. Both algorithms are subsequently compared to real-world data to optimize hyperparameters (i.e., structural model implementations) and to learn model parameters for fitting. The same optimization principles apply but the extent to which mechanistic priors are accounted for in the design of the objective function varies. Finally, validation, ideally on independently sourced data, is performed to assess the model’s performance.

**Table 1:** General conceptual differences between knowledge-driven vs. data-driven modeling.

Knowledge-driven modeling	Data-driven modeling
The current “knowledge” drives the implementation of an educated guess regarding the studied relationship. *Example: Modeling of tumor growth based on the assumption of an exponential time dependency.*	The empirical reality is approximated through a (complex) relationship. *Example: A time series of tumor growth data is approximated by a long-short-term-memory network comprising thousands of parameters.*
Data serves the purpose of validation of the implemented estimate of reality. *Example: The assumption of exponential tumor growth does not allow fitting of an observed tumor volume trajectory.*	Empirical observations dictate the extraction of information. *Example: Tumor recurrence can be predicted from imaging. Interpretability analysis revealed that tumor shape was driving this* prediction.
Generate novel hypotheses for causal mechanisms. *Example: The addition of a reasonable but previously unknown mechanism to the model enables reproduction of experimental results*.	Isolate relevant inputs from empirical datasets for a given output. *Example: Principal component analysis is used to show main factors to explain the variance in the data*
Deductive capability: extrapolation to predictions about behaviors not present in original data *Example: A model described tumor response to a single radiotherapy fraction well. Predictions of tumor response to multiple fractions are possible.*	Inductive capability: interpolation of data with limited extrapolation horizon *Example: Prediction if a tumor responds to radiotherapy (represented in the training data) - this model cannot predict if the tumor will still respond if we change the delivery (i.e. fractionation) of the treatment.*
Predict or describe dynamics of the overall system. *Example: By modeling thousands of individual cells the overall dynamic growth response of a tumor is observed.*	Infer dynamics from the overall system while governing equations and parameters are not exactly known *Example: The prediction of cell states based on environmental and transcriptomic data*
Small but specific data set is needed for validation *Example: 2-3 diffusion-weighted MRI scans suffice to fit a mechanistic tumor growth model to data of an individual patient.*	Large number of parameters (thousands, millions or more), requiring data-intensive training/fitting *Example: For the prediction of tumor response to radiotherapy, 100s of patient images were used to train a deep convolutional neural network.*
Limiting factor(s): Quality of assumptions; parameter sensitivity *Example: If the underlying assumptions do not hold up upon model fitting, the model needs to be reworked.*	Limiting factor(s): Quality and quantity of data; model structure such as choice of features (inputs) *Example: A large number of diverse training examples are needed to fit a complex architecture.*

Some aspects here are taken from Baker et al. (9).

Given these similarities and differences, it is important to account for possible challenges upon combining approaches. Model bias or conflicting information generated by addressing the same task with differently motivated approaches needs to be carefully considered. At the same time, there exists ample room to harness synergies between knowledge and data-driven modeling under the umbrella of mechanistic learning. Specifically, differences regarding data requirements, model complexity, extrapolation, and application regimes imply that a combination of both approaches may mitigate individual limitations. For example, parameters of a mechanistic mathematical model can be estimated by a deep learning algorithm from complex multi-omics data or knowledge-driven descriptions can be used to constrain the large range of possible solutions of a complex data-driven approach to a meaningful subset. In the following sections, we provide a detailed overview of how these combinations can be achieved and provide real-world application examples to motivate these.

## 3 Facets of mechanistic learning

**Figure 1:**
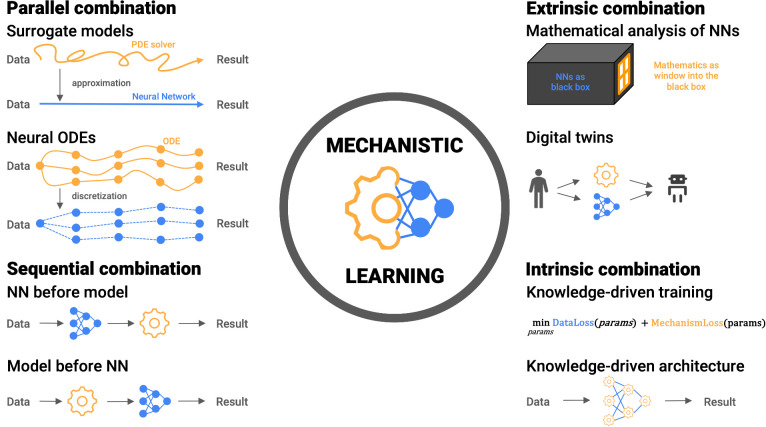
Examples of mechanistic learning structured in four combinations: Parallel combinations (top left) with examples of surrogate models and neural ordinary differential equations (ODEs). Data- and knowledge-driven models act as alternatives to complement each other for the same objective. Sequential combinations (bottom left) apply data- and knowledge-driven models in sequence to ease the calibration and validation steps. Extrinsic combinations (top right) combine knowledge-driven and data-driven modeling at a higher level. For example, mathematical analysis of data-driven models and their results or as complementary tasks for digital twins. Intrinsic combinations (bottom right), like physics- and biology-informed neural networks include the knowledge-driven models into the data-driven approaches. Knowledge is included in the architecture of a data-driven model or as a regularizer to influence the learned weights.

“Mechanistic learning” (7, 8) can take on many facets by shifting the emphasis of the “data” and “knowledge” paradigms upon model design and fitting. While a partition of mechanistic learning into simulation-assisted machine learning, machine-learning-assisted simulation, and a hybrid class for approaches falling between these definitions is intuitive at first (44), it fails to describe the variety of hybrid approaches. We suggest a more abstract classification (**Figure 1**):

- Sequential - Knowledge-based and data-driven modeling are applied sequentially building on the preceding results
- Parallel - Modeling and learning are considered parallel alternatives to complement each other for the same objective
- Extrinsic - High-level *post hoc* combinations
- Intrinsic - Biomedical knowledge is built into the learning approach, either in the architecture or the training phase

**Figure 2:**
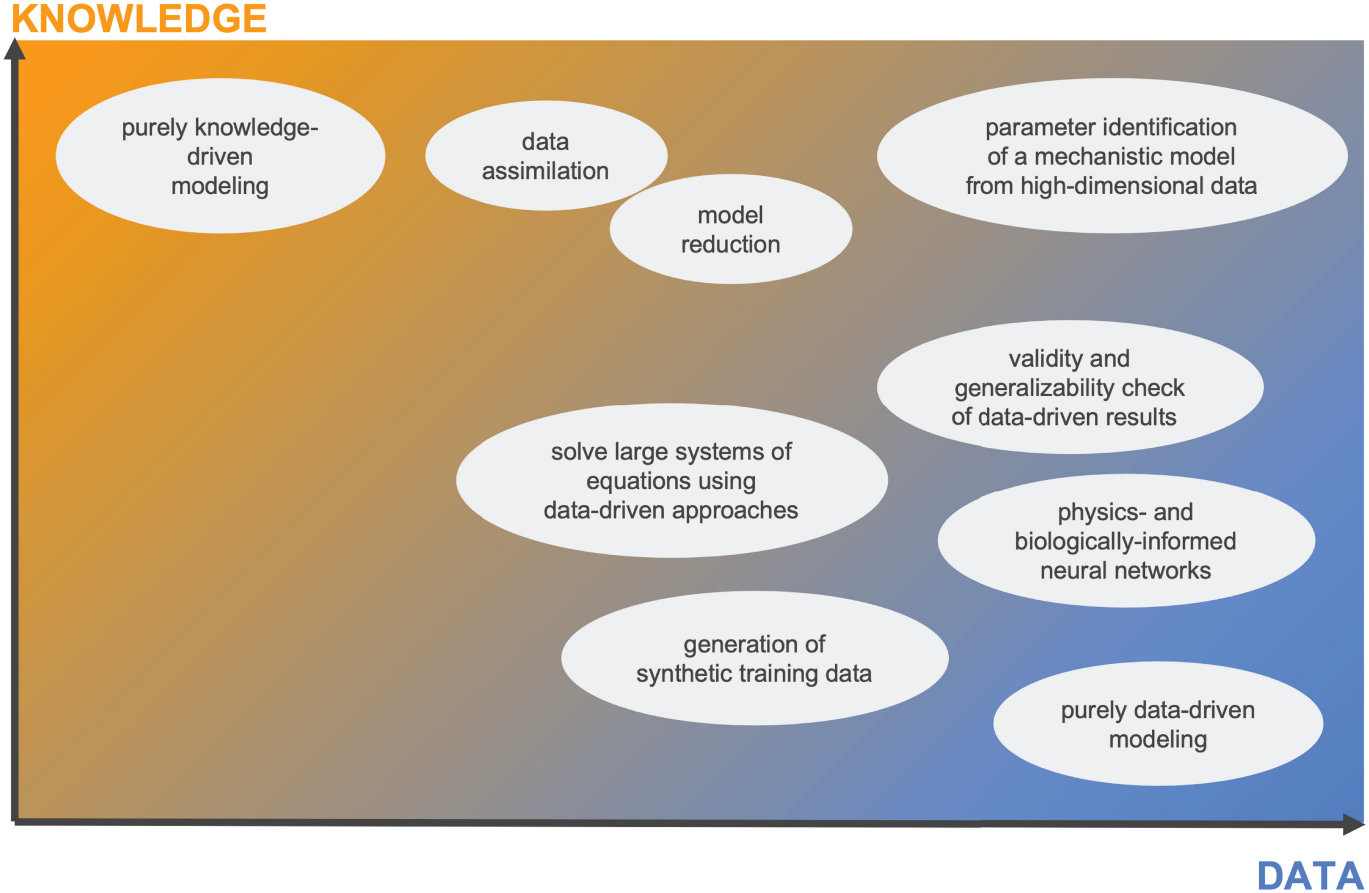
The mechanistic learning landscape shows room for the combination of data-driven and knowledge-driven modeling. We suggest that purely data-driven or purely knowledge-driven models represent the extremes of a data-knowledge surface with ample room for combinations in different degrees of synergism. Further, in the left-bottom corner with almost no data nor knowledge, any modeling or learning technique is limited.

Whereas sequential and parallel combinations make a deliberate choice of aspects of data- and knowledge-driven models to coalesce, extrinsic and intrinsic combinations actively interlace these. Thus, the complexity with respect to implementation and interpretation grows from sequential to intrinsic combinations. While most implementations readily fit into one of these four classes, we emphasize that we do not consider the combinations as discrete encapsulated instances. Instead, we view all synergistic combinations on a continuous landscape between the two extremes of purely knowledge- and data-driven models (**Figure 2**).

### 3.1 Sequential combinations

Sequential approaches harness knowledge and data-driven aspects as sequential and computationally independent tasks by disentangling the parameter/feature estimation and forecasting steps. They strive to attain mechanistic learning objectives by interlinking inputs from one approach with another. This could involve utilizing data-driven methods for estimating mechanistic model parameters or implementing feature selection in a data-driven model guided by mechanistic priors. Although sequential frameworks are straightforward to implement and interpret, often their computational demands increase significantly, taking into account both computational requirements and the limitations inherent in the individual approaches (e.g., data requirements, accuracy of prior knowledge).

#### 3.1.1 Domain knowledge to steer data-driven model inputs and architecture choices

In medical science, data availability remains a key challenge (45). However, there often exists a strong hypothesis regarding the driving features of a specific prediction task. A simple but effective means of improving the performance of data-driven algorithms is a deliberate choice of model architecture, data preprocessing, and model inputs. For example, focusing the input of a deep neural network to disease-relevant subregions of an image boosted classification performance in a data-limited setting (46), and expert-selected features were used to reduce data requirements of image processing tasks dimensionality and data requirements of image processing tasks (47). Similarly important is a deliberate choice of model architecture (48–50). For instance, while convolutional blocks are the staple for computer vision tasks, similar approaches exist for sequential data (e.g. sequence-to-sequence transformers, recurrent neural networks, or graph-based models (51, 52)). While no mechanistic modeling is conducted *per se* deliberate feature and architecture selection includes additional information. Ultimately, features can also be identified by knowledge-driven modeling (53, 54).

#### 3.1.2 Mechanistic feature engineering

Feature engineering is the process of designing input features from raw data (55). This process can be guided by a deeper understanding of the underlying mechanisms, including physical and biochemical laws or causal relationships.

Aspects of a mechanistic model can serve as input features to or outputs from machine learning models. This strategy of “mechanistic feature engineering”, was used by Benzekry et al. to predict overall survival in metastatic neuroblastoma patients (56). First, a mechanistic model of metastatic dissemination and growth was fitted to patient-specific data. Then, a multivariate Cox regression model predicted overall survival from available clinical data with or without patient-specific mechanistic model parameters. They found that including the fitted mechanistic model parameters greatly enhanced the predictive power of the regression. One problem in this truly sequential setting is that it is difficult to address uncertainty propagation. Therefore, a challenging limitation persists, as the propagation of uncertainties and prediction errors may amplify within the context of the complete framework.

#### 3.1.3 Data-driven estimation of mechanistic model parameters

A common problem in knowledge-driven modeling for longitudinal predictions is parameter identifiability and fitting given limited data and complex systems of equations. The bottleneck lies in the lack of a detailed understanding of the mechanistic relation between input data and desired output, rather than a purely computational limitation.

Similar to using mechanistic feature engineering for data-driven model inputs, data-driven approaches can also be employed to discover correlations within unstructured, high-dimensional data to provide inputs to knowledge-driven models. Depending on the specific application a range of methods are possible: imaging data are preprocessed by convolutional architectures, whereas omics data could be processed with network analysis, graph-based, or standard machine learning models. These correlations are then harnessed to predict the parameters of a mechanistic approach. Importantly, each model is implemented and trained/fitted independently, implying a high-level, yet easily interpretable combination. This sequential combination harnesses the ability of data-driven models to extract information in the form of summarizing parameters from high dimensional and heterogeneous data types. Importantly, the type of data required for such analysis needs to meet the criteria of knowledge-driven (e.g., longitudinal information) and data-driven (e.g., sufficient sample size) approaches alike - this may restrict applicability in light of limited data quality or excessive noise. Similarly, limitations such as robustness and prediction performance for the estimated parameters should be considered.

In practice, Perez-Aliacar et al. (57) predicted parameters of their mechanistic model of glioblastoma evolution from fluorescent microscopy images. This combination of models has also been suggested in the context of data-driven estimation of pharmacokinetic parameters for drugs (58). Moreover, data-driven models enable parameter inference by studying parameter dependencies of simulation results through approximate Bayesian computation (59, 60) or genetic algorithms (61).

#### 3.1.4 Data-driven estimation of mechanistic model residuals

Another sequential construct consists in using machine learning models to predict the residuals of a mechanistic model prediction. Kielland et al. utilized this technique to forecast breast cancer treatment outcomes under combination therapy from gene expression data (62). Initially, a mechanistic model of the molecular mechanisms was calibrated with cell line data to enable patient-specific predictions. Subsequently, various machine learning models were employed to predict the residuals of the mechanistic model from the available expression of more than 700 genes. While the performance of the combined strategy was comparable to using machine learning alone, it offered three advantages. First, the mechanistic model provided a molecular interpretation of treatment response. Additionally, this approach facilitated the discovery of important genes not included in the mechanistic model. Hence, this approach can potentially incorporate emerging biological knowledge and new therapeutics without additional data required for machine learning alone. Note that this sequential strategy facilitates the inclusion of both mechanistically understood features and others that may not be as clear, a common scenario in treatment forecasting.

In summary, sequential combinations are attractive due to their clear path toward implementation and interpretation with limitations due to prerequisites on data, mechanistic understanding or uncertainty propagation. While future directions may dive deeper into harnessing more complex input data (e.g. multi-omics, multimodal) for mechanistic model inputs, the technical advancement for sequential combinations remains dictated by the progress in the individual fields.

### 3.2 Parallel combinations

Parallel combinations blend advantages of purely data- or knowledge-driven models without changing the anticipated evaluation endpoint. These are alternatives for the same task as a purely data- or knowledge-driven approach and hence aspects concerning data requirements, implementation, model robustness, and performance can be compared. This makes them attractive for high-stakes decision scenarios, such as clinical application (e.g. tumor growth prediction).

#### 3.2.1 Neural networks as surrogate models

Many phenomena in oncology can be readily formulated using large systems of equations. However, solving large models comes at a high computational cost. Utilizing methods such as model order reduction aids in optimizing the computational efficiency of the solving process. This approach typically demands substantial mathematical expertise and is not suitable for time- or resource-constrained scenarios such as real-world clinical deployment. Neural networks, as universal function approximators, offer an efficient alternative. In practice, data-driven models are trained on numerical simulation results and approximate a solution to the system of equations. The inference step of the successfully trained model takes a fraction of the computational resources compared to the full mechanistic model (63, 64).

A related concept is the generation of vast amounts of “synthetic” training data (65) based on a small set of “original” data points. While synthetic training data can improve the accuracy of many learning-based systems, care needs to be taken to prevent encoding faulty concepts or misleading biases into the training data that are not present in reality (66, 67). Any uncertainty or bias introduced during the training of the synthetic data generator is inherent in the resulting samples. This limitation could easily be overlooked within downstream tasks, underscoring the importance of meticulously designing a surrogate model.

For example, Ezhov et al. (68) introduced a deep learning model performing inverse model inference to obtain the patient-specific spatial distribution of brain tumors from magnetic resonance images, addressing the computational limitations of previous partial differential equation (PDE)-based spatial tumor growth and response models. A similar brain tumor growth model based on an encoder-decoder architecture trained on 6,000 synthetic tumors generated from a PDE model (69).

#### 3.2.2 Neural ordinary differential equations — neural networks as discretized ordinary differential equations

The term “neural ordinary differential equation”, or “neural ODE” originated from the notion of viewing neural networks as discretized ODEs or considering ODEs to be neural networks with an infinite amount of layers (70–72). In that sense, the knowledge-driven approaches using ODEs and the data-driven approach using neural networks are parallel perspectives of the same concept. While not every data-driven model can be interpreted as discretized ODEs and not every question for ODEs can be answered by a discretization to a neural network, neural ODEs can often be a helpful concept to translate between knowledge- and data-driven modeling. More generally, a neural ODE can also be seen as a differential equation that uses a neural network to parameterize the vector field. As such, this approach offers advantages over neural networks, including high-capacity function approximation and easy trainability, together with the extensive available theory and tools for the numerical treatment of differential equations. In addition, the continuous-time regime of differential equations allows treating irregular time series data in a natural way (73).

Neural ODEs have already been used for a variety of tasks in oncology ranging from genome-wide regulatory dynamics (74) and breast tumor segmentation in medical images (75) to time-to-event modeling (76). Importantly, neural ODEs can generate realistic synthetic data, such as longitudinal patient trajectories. As these synthetic patient data are anonymous, regularly sampled, and complete (i.e. no missing data) they address key challenges of medical data analytics: data privacy, limited data, missing data, variable data quality, and sampling time points. Synthetic patients can be shared across institutes as high-quality samples to train large-scale models, ensuring compliance with international data privacy regulations (77).

#### 3.2.3 Learning a mechanistic model equation

While oncology research generates vast amounts of data, extracting and consolidating mechanistic understanding from data is a laborious process reliant on human experts. Symbolic regression allows for automated and data-driven discovery of governing laws expressed as algebraic or differential equations. This method finds a symbolic mathematical expression that accurately matches a dataset of label-feature pairs. Two prominent symbolic regression techniques are genetic programming-based optimization (78) and sparse regression (79). In genetic programming, closed-form expressions are represented as trees and evolved such that trees with high goodness-of-fit are selected for further exploration. In sparse regression strategies, the target expression is assumed to be a linear combination of certain “basis functions”, and L1 regularization is used to select and weight a small combination of them.

Despite remarkable success in physics (78), symbolic regression applications in oncology are still scarce. In one example, by Brummer et al. (80), sparse regression was employed to estimate a system of ODEs from *in vitro* CAR T-cell glioma therapy data. Compared to knowledge-based models, this data-driven approach offers new insights into the biological dynamics as the model form is not constrained.

However, estimating derivatives from high noise and sparse longitudinal measurements, like many from clinical oncology, remains challenging. Several groups have used variational formulations of ODEs and PDEs in the optimization step without relying on estimating derivatives from noisy and sparse data (81–83). Bayesian approaches applied to genetic programming have also proven successful in situations where existing non-Bayesian approaches failed (84). Other promising directions in oncological research are Koopman theory (85) and the universal differential equation framework (86), where neural networks are used to model all or part of a differential equation, facilitating the discovery of governing equations, or parts of them, in cases where data are limited.

### 3.3 Extrinsic combinations

Extrinsic combinations make use of both mechanistic and data-driven approaches to address different aspects of the same problem or to post-process the output of a data-driven implementation.

#### 3.3.1 Digital twins

Originating from analogies in manufacturing and engineering, the concept of digital twins (87–89) has recently gained interest in the oncology community. A digital twin is an *in silico* patient “twin” that recapitulates important patient characteristics and is used to simulate alternative treatment strategies and forecast disease progression (90). In the context of precision medicine, this implies that alternative treatment scenarios are simulated with the digital twin to select an optimal strategy. Hence, predictive modeling of longitudinal information regarding the expected patient trajectory is provided. The computational framework behind the digital twin can be based on mechanistic, data-driven, or a combined set of algorithms. We highlight the potential of combining mechanistic and data-driven modeling as side-by-side tasks, covering different aspects of one unifying digital twin.

Typically, for mechanistic digital twins, a mathematical framework describes the dynamics of tumor size, morphology, composition, and other biomarkers (91). The data-driven analogy is represented by machine learning algorithms, e.g., k-nearest neighbors but also more advanced architectures, to provide a prediction of the endpoint of interest based on established databases (92, 93). Both knowledge- and data-driven models enable the real-time adaptation of treatment protocols by simulating a range of scenarios. Importantly, harnessing the strengths of each method should be considered for optimal results. For instance, a data-driven prediction task could inform on patient subgrouping and identify likely outcomes, whereas mechanistic modeling would explore personalized treatment alternatives. Generally, digital twins can also serve as “virtual controls” to benchmark the efficacy of the patient’s current treatment regimen (94, 95). Wu et al. provide an in-depth review regarding the specific application example of digital twins for oncology applications including a mention of the roles of data-driven image analysis and knowledge-driven modeling. The trade-off between application focus and computational complexity of a digital twin has to be considered in light of the data available which may restrict the feasible complexity and performance. Limitations, such as the requirement for longitudinal data, the complexity of mid-treatment adjustment in clinical settings, and the overall complexity regarding a high-stakes decision process need to be accounted for (89).

#### 3.3.2 Complementary postprocessing: mathematical analysis of data-driven models and data-driven analysis of mathematical simulations

Data-driven approaches are trained to optimize a performance metric, but performance alone is not driving a model’s application in (clinical) practice. Here, quantification of the uncertainty of model results, model robustness, as well as interpretability to explain why a model arrived at a certain conclusion are equally important (96). These questions are usually studied under the term explainable AI; for a survey we refer to Roscher et al. (97). Progress in advanced explainable AI dictates a mechanistic interpretation of a model’s decision-making process (98).

Addressing many of the questions related to deep learning is only possible using mathematical methods, i.e., challenges in the field of data-driven models are transformed to mathematical conjectures that are subsequently (dis)proven. This approach ensures that the results generated by models are mathematically reliable and transparent and thus better suited for clinical implementations.

Numerous examples underscore this point and provide motivation for employing intricate architecture designs based on mathematical formulations. A specific instance involves learning a specialized representation that elucidates cancer subtyping from multi-omics inputs, including transcriptomic, proteomic, or metabolomic data (77).

Data assimilation techniques bridge numerical models and observational data through optimization of starting conditions. Typical examples are Kalman or particle filter methods (99, 100), which can improve the accuracy of numerical predictions. For the interpretation and validation of simulation results, tools from data-driven modeling can be used to detect patterns in simulations (101). This approach is already performed in research fields outside the oncology domain (102). A prime example is the post-processing of complex numerical weather forecasting predictions using deep learning to boost overall performance (103, 104). Within oncology applications, machine learning and Bayesian statistics have also been used for uncertainty quantification which is important for clinical translation (105–107).

### 3.4 Intrinsic combinations

This combination incorporates a mechanistic formulation within a machine learning model either upon training as a contribution to the formulated objective function or *a priori* as a way of choosing the architecture of the data-driven model. As such, these are densely interconnected combinations.

#### 3.4.1 Regularizing the loss function using prior knowledge

Mechanism-informed neural networks such as physics-informed neural networks (PINNs) (108, 109) use mechanistic regularization upon training, i.e., equation-regularization, by guiding the possible solutions to physically relevant ones. The loss function combines performance loss with a regularization term assessing the deviation from a predefined set of equations. This approach reduces overfitting and ensures physically meaningful predictions. The final neural network will not satisfy the equations exactly but approximate them for the areas where training data is available. PINNs can be valuable for deciding whether an equation can be used to describe data by considering several related equations as regularizers.

Equation-regularization has previously been shown to enhance both the performance and interpretability of data-driven architectures. In the context of oncology, one example includes the modeling of tumor growth dynamics (110). Ayensa-Jiménez et al (111) used physically-guided NNs with internal variables to model the evolution of glioblastoma as a “go-or-grow” process given constrained resources such as metabolites and oxygen. The model-free nature of their approach allows for the incorporation of data from various boundary conditions and external stimuli, resulting in accurate tumor progression predictions even under different oxygenation conditions.

#### 3.4.2 Incorporating knowledge into the machine learning model architecture

Rather than optimizing a network architecture through regularization, biology-informed neural networks constrain the model architecture to biological priors from the start. Typically in the context of network analysis, biological priors such as known interactions between genes and/or transcription factors are translated to nodes and edges in a graph (112, 113). The network is constrained to an established connectivity profile which greatly reduces the model complexity compared to a fully connected network. Similar to transfer learning where a different data-rich scenario is used to pretrain a model prior to refining specific weights on the limited target data, this approach uses expert insight to preset connections and weights. Lagergren et al. (114) proposed biology-informed neural networks that learn the nonlinear terms of a governing system, eliminating the need for explicitly specifying the mechanistic form of a PDE as is the case for PINNs. They tested their approach on real-world biological data to uncover previously overlooked mechanisms. Another example is given by Przedborski et al. (115) who used biology-informed neural networks to predict patient response to anti-PD-1 immunotherapy and present biomarkers and possible mechanisms of drug resistance. Their model offers insights for optimizing treatment protocols and discovering novel therapeutic targets. Indeed, this approach has found several applications, e.g., for the prediction of prostate cancer (112) and drug discovery (116). Despite similar naming conventions, biology- and physics-informed neural networks refer to distinct approaches. The latter distinguishes itself by integrating biological realism and enhancing interpretability for applications that predominately rely on multi-scale, multi-source data (such as omics). However, profound insight regarding the formulated biological process is indispensable. PINN applications regularize, i.e. do not strictly constrain implying more flexibility yet less interpretability.

Finally, in the context of generative approaches, differential equations have previously been incorporated into (deep) neural networks through variational autoencoders. While current examples were obtained from medical applications other than oncology (117, 118), they represent elegant solutions to allow for dynamic deep learning despite limited data, given careful hyperparameter tuning.

#### 3.4.3 Hierarchical modeling

Hierarchical nonlinear models, also referred to as nonlinear mixed effects models, are a widely used framework to analyze longitudinal measurements on a number of individuals, when interest focuses on individual-specific characteristics (119). For instance, early in drug development, pharmacokinetics studies are carried out to gain insights into within-subject pharmacokinetics processes of absorption, distribution, and elimination (120). Typically, a parametric nonlinear model describing drug concentration change over time (individual-level model) is coupled with a linear model describing the relation between pharmacokinetic parameters and individual features (population-level model). One of the simplest population-level models is the random intercept model, which models individual parameter values as normally distributed around a typical value. This enables information sharing through each individual’s contribution to determine the typical value, while simultaneously allowing individual parameters that match the observed measurements. Moreover, in contrast to the sequential approach (section 3.1.3), hierarchical models allow for the propagation of uncertainty between the individual-level and population-level models. Applications in oncology range from tumor growth (121) to mutational dynamics in circulating tumor DNA (122) or metastatic dissemination (123).

Interestingly, hierarchical models have the potential to benefit from more sophisticated data-driven approaches to integrate high-throughput data, such as omics or imaging (8). This can be done by changing the linear covariate model with more complex machine learning algorithms able to capture complex relations between the parameters of the individual-level model and the high dimensional covariates (124, 125), and/or by using Bayesian inference (38).

## 4 Conclusion and perspective

Recently, machine and deep learning have become ubiquitous given their indisputable potential to learn from data (126). However, it is evident that medical applications, especially in oncology, are currently constrained by the extent and diversity of available data. Moreover, clinical translation involves high-stakes decisions that need to be backed up by evidence. The oncology field must address the critical challenges of limited data availability, model transparency, and complex input data. To overcome these bottlenecks, we need data-efficient, comprehensible, and robust solutions. Despite the growing interest in mechanistic mathematical modeling for medical applications, the success and opportunity of data-driven models must be taken into account. Strategically integrating knowledge- and data-driven modeling in mechanistic learning represents a logical progression to tackle the challenges in mathematical oncology. It aims to facilitate accurate, personalized predictions, leading to a more comprehensive understanding of cancer evolution, progression, and response.

Here, we identified opportunities for synergistic combinations and provided a snapshot of the current state-of-the-art for how such combinations are facilitated for oncology applications. We highlighted similarities in the mathematical foundation and implementation structure of optimization processes and pointed out differences with respect to data requirements and the role of knowledge and data in these approaches. It is important to structure the growing landscape of models at the interface of data- and knowledge-driven implementations. We hence propose systemizing combinations in four general categories: sequential, parallel, intrinsic, and extrinsic combinations. While sequential and parallel combinations are intuitive and easily implemented, intrinsic and extrinsic combinations incorporate a stronger degree of interlacing that requires a deeper understanding of both data science and mathematical theory. The choice of analysis tool should always keep in mind the quality, size, and type of data and knowledge in light of the underlying research question. An intentional combination of machine learning and mechanistic mathematical modeling can then leverage the strengths of both approaches to tackle complex problems, gain deeper insights, and develop more accurate and robust solutions. Mechanistic learning can take on many facets and is foreseen to grow in importance in the context of mathematical oncology with a particular focus on explainable AI, handling of limited data (e.g. efficient architecture design, data augmentation), and generation of precision oncology solutions. In this review, we discussed only the core concepts. Given the fluid boundaries between data- and knowledge-driven models and in light of the variety of approaches within each of these domains, an exhaustive listing of all combinations is infeasible. However, several future directions stand out. For instance, hybrid modeling with Bayesian statistics, deep generative approaches, or specific training regimes, including semi-supervised (contrastive) or reinforcement learning, are worth mentioning. Finally, despite the positive notion regarding mechanistic learning, certain limitations persist within both separate and combined approaches. Specifically ethical considerations should be addressed. These may arise from data privacy, algorithmic bias, or the clinical implementation of hybrid models.

Finally, with this work we strive to motivate a more active exchange between machine learning and mechanistic mathematical modeling researchers given the many parallels in terms of methodologies and evaluation endpoints, and the powerful results produced by mechanistic learning.

## Author contributions

JM: Conceptualization, Formal analysis, Visualization, Writing – original draft, Writing – review & editing. CJ: Conceptualization, Supervision, Writing – review & editing. PM: Conceptualization, Supervision, Writing – review & editing. AK: Conceptualization, Writing – original draft, Writing – review & editing. SB: Conceptualization, Formal analysis, Project administration, Writing – original draft, Writing – review & editing.
